# Ho^3+^ codoping of GGAG:Ce: a detailed analysis of acceleration of scintillation response and scintillation efficiency loss†

**DOI:** 10.1039/d4ra02866j

**Published:** 2024-07-22

**Authors:** Juraj Páterek, Pavel Boháček, Bohumil Trunda, Vladimir Babin, Richard Švejkar, Karel Jurek, Jan Rohlíček, Martin Nikl

**Affiliations:** a Institute of Physics, Czech Academy of Sciences Cukrovarnická 10 Prague Czech Republic paterek@fzu.cz nikl@fzu.cz; b Faculty of Nuclear Sciences and Physical Engineering, Czech Technical University in Prague Břehová 7 Prague Czech Republic

## Abstract

In this study, we investigate the effects of Ho^3+^ codoping on the luminescence and scintillation properties of GGAG:Ce, with a particular focus on timing properties and scintillator efficiency. The research reveals that Ho^3+^ codoping and subsequent resonant energy transfer from Ce^3+^ to Ho^3+^ can significantly reduce the 5d_1_ excited state decay time of Ce^3+^ and shorten scintillation pulses of GGAG:Ce registered by using photomultipliers, although this reduces scintillator efficiency as well. The study presents a detailed analysis of the loss of scintillator efficiency due to Ho^3+^ codoping, identifying the most significant loss pathways and estimating their impact. The findings suggest that Ho^3+^ codoping is an effective method for accelerating the scintillation response of GGAG:Ce. Furthermore, the study presents a high level of consistency of the Ce^3+^ kinetics with the Inokuti–Hirayama model and with results obtained in the previous studies on similar systems, demonstrating the predictability of the effect of RE^3+^ codoping on scintillator properties.

## Abstract 2

In this study, we investigate the effects of Ho^3+^ codoping on the luminescence and scintillation properties of GGAG:Ce, with a particular focus on timing properties and scintillator efficiency.

## Graphical abstract

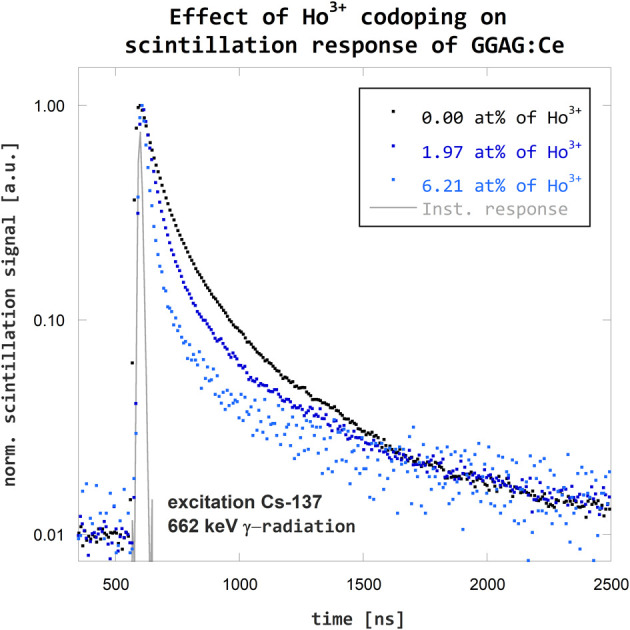

## Introduction

Codoping with trivalent rare-earth ions (RE^3+^) was proven to be an effective method for shortening of the activator decay time and scintillation response in Ce^3+^/Pr^3+^ activated garnet scintillators. This has been demonstrated in our previous studies on Er^3+^ and Ho^3+^ codoping of YAG:Ce (Ce^3+^ doped yttrium aluminum garnet),^1,2^ Ho^3+^ codoping of LuAG:Pr (Pr^3+^ doped lutetium aluminum garnet)^3^ and other RE^3+^ codoping of garnets.^4,5^

**Fig. 1:**
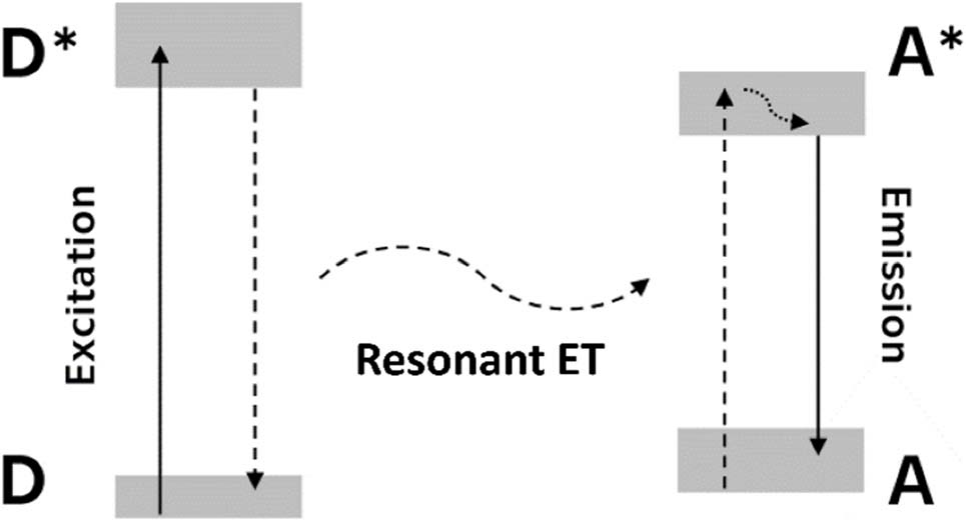
Schematic of RET mechanism directed from donor (D, asterisk indicates excited state) to acceptor center (A). Radiative transitions – donor excitation and acceptor emission – drawn with solid line, relaxation to lower energy drawn with dotted line, resonant ET drawn with dashed line.

The acceleration of the activator decay is enabled by resonant energy transfer (RET). This effect involves transition of one of the centers (donor) to a lower energy state and simultaneous promotion of another distant center (acceptor) to a higher energy state. The mechanism of RET is depicted in Fig. 1.

Unlike reabsorption, where energy emitted in the form of a photon by one center and absorbed by another, the RET does not include the formation of a photon. It is driven by multipole electro–magnetic interaction. RET is enabled between luminescence centers in resonance, *i.e.* centers whose emission and absorption spectra overlap. Rate of RET is proportional to the overlap of the emission spectra of the donor *f*_D,em_ and absorption spectra of the acceptor *f*_A,abs_ and is inversely proportional to the power of the distance between ions *R*1
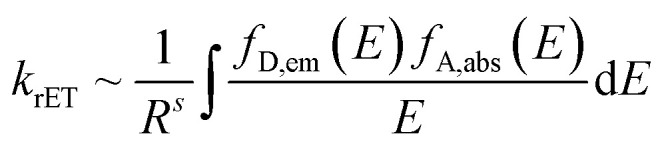
where *s* is set to 6, 8 or 10 for dipole–dipole, dipole–quadrupole and quadrupole–quadrupole interaction of the ions, respectively. For our application, it is favorable to restrict RET between ions only to one direction. This can be ensured by selecting center with large Stokes shift as the donor center and/or center with fast relaxation of the excited state to energetically lower states as acceptor. Then RET contributes to the total decay rate *k*_tot_ of the donor center as follows

2 *k*_tot_ = *k*_inh_ + *k*_rET_

where *k*_inh_ is inherent decay rate of the donor and *k*_rET_ is decay rate due to RET towards acceptor, and thus, increase its decay rate, shorten its decay time, and eventually accelerates the scintillation response of the material they are hosted in.

The downside of the RE^3+^ codoping is reduction of the donor emission and subsequently scintillator efficiency. Same as shortening of the donor decay time, reduction of the activator emission is caused by RET, hence inevitable. In this sense, the Ho^3+^ acceptor can be considered a killer center for Ce^3+^ 5d → 4f emission. Then, using the model for number of UV/visible photons *N*_ph_ generated per energy of incident radiation *E* derived in^6–8^3
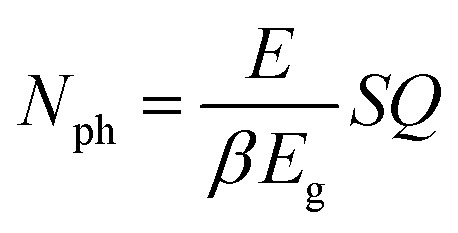
where *E*_g_ stands for the material band gap, *S* and *Q* are quantum coefficients related to efficiency of the transport and luminescence stages and *β* is a phenomenological parameter found to be between 2 and 3 for most materials. See Fig. 1 in ref. 9 for schematic explanation of the role of *S*, *Q* quantum coefficients in the scintillation mechanism. Ho^3+^ content will affects the coefficient *Q* that reflects the contribution of killer centers.^9^ The coefficient *Q* is inversely proportional to Ho^3+^ content, *i.e.* the greater the content of the acceptor centers the lesser the *Q* coefficient, and eventually the lesser the number of photons emitted by Ce^3+^ center and scintillator efficiency.

Ho^3+^ ions have been found to be particularly effective acceptor codopants for Ce^3+^ and Pr^3+^ activated garnets as they enable acceleration of the Ce^3+^/Pr^3+^ decay time due to RET without introducing any additional signal to the detection spectrum or introducing slow components of light, see ref. 1 and 3.

Multiple RE^3+^ ions other than Ho^3+^ could be used as acceptor in pair with Ce^3+^ donor in GGAG, *i.e.* would reduce the decay time of the 5d state of Ce^3+^ due to RET. According to ref. 10 and 11, they are Pr^3+^, Nd^3+^, Pm^3+^, Sm^3+^, Eu^3+^, Tb^3+^, Dy^3+^, Er^3+^ and Tm^3+^. However, as shown in our previous studies for Er^3+^,^1,2^ Dy^3+^,^3^ and Nd^3+^ (ref. 4) and studies of other authors for Sm^3+^,^12^ Eu^3+^ and Tb^3+^,^13^ and Tm^3+^ (ref. 14) unlike Ho^3+^ all of them have parity-forbidden 4f → 4f emission positioned in the range of Ce^3+^ emission which would introduce slow components into detectable emission when using common photomultipliers or even Si-based semiconductor photodetectors. This is counterproductive to the effect of shortening of the scintillation response and would unavoidably lead to impaired timing properties of the scintillator. The situation is specific for Pr^3+^. This ion is typically used as an activator of garnet scintillators for its fast 5d → 4f emission positioned in UV range but emits also between 480 and 650 nm due to 4f → 4f transitions.^15^ However in case of Pr^3+^ codoping of GGAG:Ce, its 5d → 4f emission transition would transfer energy into the Gd sublattice which diminishes fast scintillation response,^16^ while, the 4f → 4f transitions would remain active and introduce slow light to detectable signal same as the RE^3+^ ions above. Pm^3+^ is not considered due to low practical use of this element due to absence of stable isotope.

This study builds on upon these previous findings by examining Ho^3+^ codoping of GGAG:Ce,Mg (gadolinium aluminum gallium garnet doped with Ce^3+^ and Mg^2+^). GGAG:Ce is a representative of multicomponent garnets compounds of general chemical formula of the host (Gd,Lu,Y)_3_(Al,Ga)_5_O_12_. They have been reported firstly in the ceramic form^17,18^ and their enormously high scintillation light yield up to 50 000 phot per MeV and excellent energy resolution of 4.8%@662 keV immediately interested researchers in scintillator field. These materials can be prepared also in single crystal form, most frequently reported by Czochralski technique where even 4 inch diameter large crystals have been achieved.^19^ Another preparation techniques, *e.g.* floating zone has also been reported.^20^ High entropy alloys in multicomponent garnet family were also studied which was fueled by an interest to find unusual stable compositions with unique properties^21^ and combinatorial research strategy was applied as well.^22^ Effects of composition and growth parameters on phase formation in multicomponent aluminum garnet crystals was systematically studied.^23^ Luminescence investigation focused on the interplay between the Ce^3+^ luminescence center and the host due to decreasing ionization barrier of the Ce^3+^ 5d_1_ excited state,^24,25^ traps states acting in scintillation mechanism were studied by thermoluminescence techniques.^26^ In the study of scintillation characteristics special attention was paid to stabilization of Ce^4+^ by stable divalent dopants as Mg^2+^ or Ca^2+^ which creates new fast radiative recombination pathway at Cerium centers and accelerates noticeably the scintillation response.^27–30^ Other codopants were studied for this purpose as well.^31^ Dependence of the bandgap value on the host composition was also studied^32,33^ and garnet compounds luminescence and scintillation characteristics were reviewed in ref. 34. The application potential of multicomponent garnets for fast timing application in medical imaging and high energy physics was evaluated in ref. 35 and 36.

In this paper, to better understand the mechanisms behind the acceleration of scintillation response due to RE^3+^ codoping, its benefits and drawbacks, we examine not only direct effects of Ho^3+^ codoping on scintillation characteristics of GGAG:Ce,Mg like decay time and light yield (LY), but also investigate the effect of Ho^3+^ codoping on specific stages of scintillation mechanism in detail. Further, findings obtained in this, and previous studies are compared and discussed and build up the picture of the RE^3+^ codoping for modification of scintillation properties as a method in general.

## Experimental methods

Electron probe microanalysis (EPMA) analysis was performed using JEOL JXA-733 microprobe. Crystal structure was examined by powder X-ray diffraction pattern analysis (XRD) measured at powdered small piece of the samples using the Bragg–Brentano focusing configuration on the powder diffractometer Empyrean of PANalytical (λCu, Kα = 1.54184 Å) that was equipped with a fixed divergent slit and PIXcel3D detector. 120 minutes long measurements were made from 4 to 100° 2*θ* with 0.013° step size and 300 s per step. Absorption spectra were measured with a Shimadzu 3101 PC spectrometer. A Horiba Jobin Yvon 5000M spectrofluorimeter equipped with a TBx-04 photon counting detector was used for the steady-state spectral measurements and measurement of the photoluminescence decay. Excitation was performed with a Seifert tungsten X-ray tube (40 kV, 15 mA) and an Heraus deuterium lamp for radioluminescence and photoluminescence spectroscopy, respectively. All the spectra were corrected for the spectral distortions of the setup. The photoluminescence decay kinetics of the Ce^3+^ center were measured by a time-correlated single photon counting method^37^ with a Horiba NanoLED nanosecond excitation source. The scintillation decay curves were obtained with use of ^137^Cs γ-ray excitation, Tektronix TDS3052C digital phosphor oscilloscope, and a fast photomultiplier Hamamatsu R7207-01 working in current regime. Amplitude spectra for LY measurement^38,39^ were obtained with a shaping time of 1 μs, ^137^Cs γ-ray excitation, and a hybrid photomultiplier Photonis PP0475B. All before mentioned measurements were performed at room temperature. Thermally stimulated luminescence (TSL) was measured in range 77–700 K with heating rate 0.1 K s^−1^. Temperature of the sample was regulated with Janis N2 VPF-800 cryostat. Initially, sample was irradiated with X-ray (40 kV, 15 mA) for 10 minutes at 77 K. Then, spectrally unresolved TSL glow curves were recorded using IBH Scotland TBx-04 photomultiplier in the photon counting mode and 1 s sampling rate. Photoluminescence and scintillation decay kinetics were analyzed using iterative least-square re-convolution method^40^ and Python packages LMfit^41^ and SciPy.^42^

## Results and discussion

### Preparation and composition analysis of the samples

**Fig. 2:**
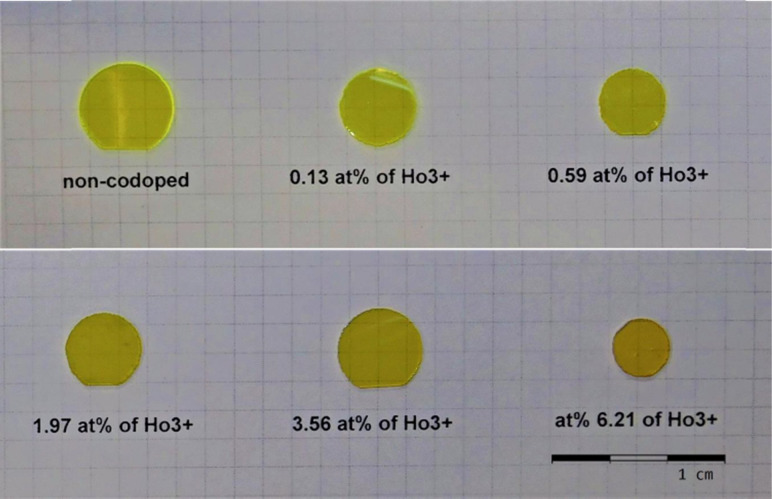
Photography of GGAG:Ce crystals codoped with various concentrations of Ho^3+^. The reddish tint is due to Ho^3+^ codoping of the crystals.

A set of six GGAG crystals was prepared by the Czochralski method^43^ from melts with starting compositions Gd_2.9844−*x*_Ce_0.015_Mg_0.0006_Ho_*x*_Ga_2.7_Al_2.3_O_12_, with *x* = 0.00, 0.015, 0.030, 0.045, 0.090 and 0.150. Platelets of the thickness of 1 mm were prepared from the tip parts of the crystal's, see Fig. 2. As the Mg^2+^ codoping has no effect on the Ce^3+^–Ho^3+^ energy transfer process, the materials will be referred only as GGAG:Ce, or Ho^3+^ codoped GGAG:Ce in the texts below, even though they contain the Mg^2+^ dopant as well.

Actual concentrations of the Ce^3+^ and Ho^3+^ dopants were determined using the EPMA and absorption spectroscopy. At first, the concentration of Ce^3+^ and Ho^3+^ was measured using EPMA for sample *x* = 0.045 providing 0.18 and 1.97 at% (expressed as a percentage of Gd atoms replaced by the dopant), respectively. Concentration of the dopants in the remaining samples was determined using the integrals of absorption peaks,^44^ namely the 4f → 5d_1_ transition for Ce^3+^ (390–510 nm), ^5^I_8_ → ^5^S_2_ + ^5^F_4_ (520–561 nm) and ^5^I_8_ → ^5^F_5_ (626–674 nm) transitions for Ho^3+^. Concentrations of Ce^3+^ dopant were found at the value of 0.18 ± 0.02 at% for all the samples. For Ho^3+^ the values of 0.00, 0.13, 0.59, 1.97, 3.56 and 6.21 at% were found. Concentrations of Mg^2+^ were too low to be measured by EPMA, *i.e.* lower than 0.01 at%.

XRD analysis confirmed single garnet phase in all the samples (see example in Fig. S2†) with the exception of the highest Ho concentration one, *i.e.* GGAG:Ce with 6.21 at% of Ho^3+^, see Fig. S1 in ESI.† In this sample, the secondary phase of the same garnet structure with a little bigger lattice constant was found. Its content (estimated from XRD analysis) is less then 5wt%. EPMA analysis of the secondary phases islands, see Fig. S3,† showed it is most probably due to reduced content of Ho^3+^ in the secondary phase.

Given the volume of the secondary phase in GGAG:Ce with 6.21 at% of Ho^3+^ its effect on the studied energy transfer phenomena is considered negligible. Further details on XRD and EPMA analysis are provided in ESI.†

### Acceleration of Ce^3+^ decay time and scintillation properties due to Ho^3+^ codoping

**Fig. 3:**
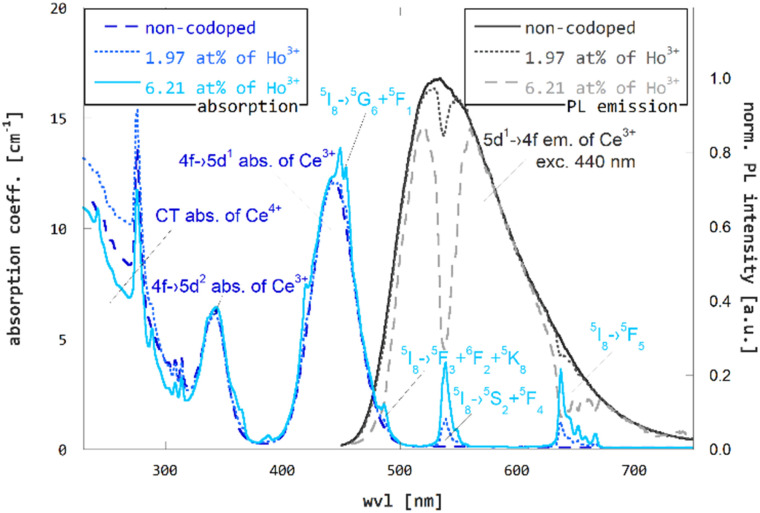
Photoluminescence (excitation to 440 nm) and absorption spectra of non-codoped GGAG:Ce and Ho^3+^ codoped GGAG:Ce shows the spectral overlap.

The effect of Ho^3+^ codoping of GGAG:Ce was examined using multiple spectroscopic methods. Results of the experiments and discussion of the findings are described in the following paragraphs. First, overlap of the Ce^3+^ emission and Ho^3+^ absorption spectra, that is a prerequisite for RET, were studied using photoluminescence steady-state spectroscopy and absorption spectroscopy. Absorption and photoluminescence spectra (excited by 440 nm) of GGAG:Ce and Ho^3+^ codoped GGAG:Ce crystals are shown in Fig. 3. The non-codoped GGAG:Ce crystal shows typical absorption bands of Ce^3+^ allowed 4f → 5d_1_ and 4f → 5d_2_ transitions at 440 and 340 nm, a wide absorption band below 340 nm induced by charge transfer (CT) absorption band of Ce^4+^, that is induced by Mg^2+^ codoping in Ce^3+^ activated garnets^45^ and absorption lines of parity forbidden 4f → 4f transition of Gd^3+^ at around 275 and 310 nm.^46^ The same absorption patterns are observed in Ho^3+^ codoped crystals as well. In addition to that, multiple sets of narrow absorption lines of parity forbidden 4f → 4f transitions from Ho^3+^ ground state ^5^I_8_ to ^5^G_6_ and ^5^F_1_ around 449 nm, ^5^F_3_ and ^5^F_2_ and ^3^K_8_ around 486 nm, ^5^S_2_ and ^5^F_4_ around 538 nm and ^5^F_5_ around 636 nm excited states can be observed. Multiple Ho^3+^ sets of absorption lines are located below 440 nm as well. For more detailed information on UV/VIS spectrum refer to ref. 47, which reports optical transitions of Ho^3+^ in structurally similar YAG. Photoluminescence spectra of both non-codoped and Ho^3+^ codoped GGAG:Ce are dominated by wide Ce^3+^ 5d → 4f emission band ranging between 450 and 720 nm. In line with,^47^ that states the emission of Ho^3+^ in garnet matrix is positioned in the IR spectrum, no Ho^3+^-related emission is observed in the UV/VIS region. Ce^3+^ emission bands in Ho^3+^ codoped GGAG:Ce crystals are deformed due to re-absorption of emitted light by overlapping Ho^3+^ absorption lines. The same spectral overlap fulfills the prerequisite for RET.

**Fig. 4:**
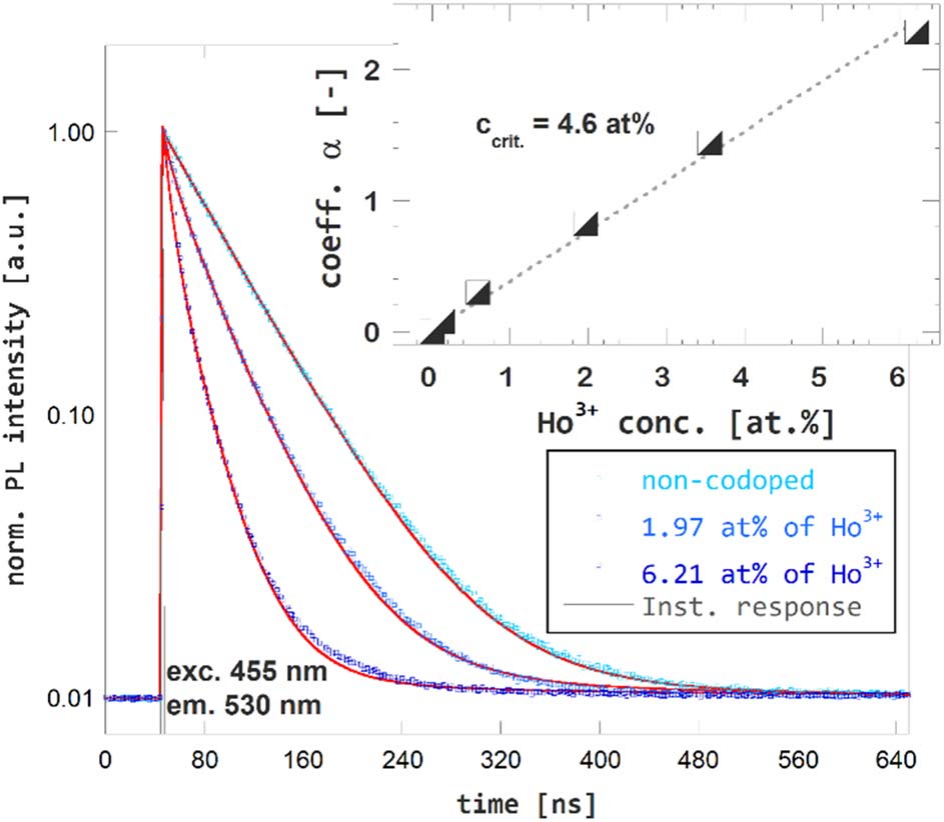
Photoluminescence decay kinetics of Ce^3+^ (excitation 455 nm, emission 530 nm) in non-codoped GGAG:Ce and Ho^3+^ codoped GGAG:Ce. The red lines show the IH model fit of the data.

The time-resolved PL spectroscopy of Ho^3+^ codoped GGAG:Ce crystals was used to investigate changes of the Ce^3+^ decay kinetics due to Ho^3+^ codoping and related RET, see Fig. 4. Time-resolved PL spectra of Ce^3+^ decays with excitation to 455 nm and emission 530 nm were recorded and reveals substantial acceleration of Ce^3+^ decay in Ho^3+^ codoped and the fact the acceleration is proportional to Ho^3+^ content. The acquired decay curves were fitted to Inokuti–Hirayama (IH) model for donor luminescence kinetics. Assuming homogenous distribution of the donor and acceptors centers through the crystal, decay kinetics of the donor center *I*(*t*) will obey the following4
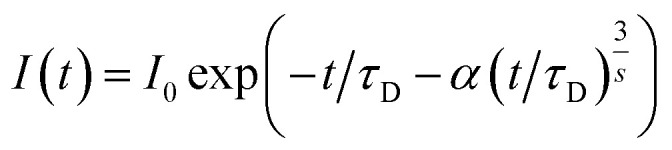
where *I*_0_ is magnitude of the decay curve, *τ*_D_ is the inherent decay time of the donor center, *s* is the parameter related to order of the multipole interaction equal to 6, 8 or 10 for dipole–dipole, dipole–quadrupole or quadrupole–quadrupole interaction, respectively and *α* is coefficient related to rate of the ET defined as 
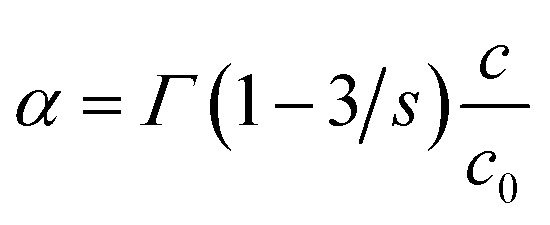
, where *Γ*(*x*) is the gamma function, *c* is the concentration of the acceptor center and *c*_0_ is critical concentration of the acceptor, *i.e.* the concentration of acceptor that yields rate of RET equal to the rate of the inherent decay rate of the donor, *i.e. k*_rET_ = *k*_inh_. The best match was achieved for *s* = 6, which refers to dipole–dipole interaction between Ce^3+^ and Ho^3+^. In line with presumptions of Inokutu–Hirayama model, parameter *α* is proportional to Ho^3+^ content, see the inset of Fig. 4. Linearity of the relation between parameter *α* and Ho^3+^ content was used to determine the critical concentration of Ho^3+^ in GGAG:Ce to 4.6 at%. The calculated 1/*e* decay time of Ce^3+^ center was shortened from 56 ns for the non-codoped GGAG:Ce to 8 ns for the GGAG:Ce codoped with 6.2 at% of Ho^3+^. Refer to Table 1 for all calculated 1/*e* decay times.

**Table 1:** Summary of photoluminescence and scintillation properties of Ho^3+^ codoped GGAG:Ce in relation to Ho^3+^ content. PL an SC *τ*_1/*e*_ stands for 1/*e* decay time of Ce^3+^ and scintillation decay, respectively, Rel. PL and SC *τ*_1/*e*_ for relative change of decay times, Rel. LY for relative LY and the last columns lists relative overall efficiency. All relative values are compared to that of the non-codoped GGAG:Ce

Ho^3+^ conc. [at%]	PL *τ*_1/*e*_ [ns]	Rel. PL *τ*_1/*e*_ [%]	SC *τ*_1/*e*_ [ns]	Rel. SC *τ*_1/*e*_ [%]	Rel. LY [%]	Rel. eff. [%]
0.00	56.1	100	90.3	100	100	100
0.13	51.9	92	105.5	117	98	111
0.59	41.8	74	82.3	91	75	89
1.97	25.2	45	53.1	59	46	59
3.56	14.7	26	36.4	40	28	40
6.21	7.9	14	25.2	28	15	22

In the next paragraph, a comparison of the Ho^3+^-codoping of GGAG:Ce and YAG:Ce grown by edge-defined growth method, that was investigated in our previous study,^1^ will be discussed. As the both matrices (GGAG and YAG) are structurally very similar and the same donor–acceptor pair was used in the studies analogical effects of the Ho^3+^-codoping are expected. In both cases, RET is enabled by overlap of the wide Ce^3+^ 5d → 4f emission band and Ho^3+^ absorption lines related to parity-forbidden 4f → 4f transitions and a good match of Ce^3+^ PL kinetics with IH model was achieved. The Ce^3+^–Ho^3+^ interaction is of dipole–dipole type, in both systems. The critical concentration of Ho^3+^ in GGAG:Ce was found just slightly higher when compared to 4.4 at% found for Ho^3+^ in YAG:Ce. The difference in critical concentration can be accounted by either of two following explanations or their combination. First, the lattice parameters increase when Y and Al are substituted by Gd and Ga, respectively – lattice parameter increase from 12 to 12.21 and 12.55 Å for Y_3_Al_5_O_12_, Gd_3_Al_5_O_12_ and Gd_3_Ga_5_O_12_, respectively.^48^ Hence, higher content of Ho^3+^ acceptor is required to achieve the mean distance between donor–acceptor pairs to be critical distance in GGAG:Ce. Second, the actual and nominal values of Ho^3+^ content in Ho^3+^ codoped YAG:Ce crystals may vary, as the nominal values refer to content of Ho^3+^ in melt.

Consistency of results observed in structurally similar, but not identical GGAG and YAG matrices, grown by different methods and a good match of the measured PL decay curves with the IH model in both cases make the Ho^3+^-codoping well predictable method for tuning of Ce^3+^ luminescence kinetics. It is necessary to say, the application of the studied method is not restricted to RE^3+^ codoping of Ce^3+^ and Pr^3+^ activated garnets, but can be universally applied to any family of matrices and combination of donor–acceptor pairs. The only condition is the resonance between the donor and acceptor transitions.

**Fig. 5:**
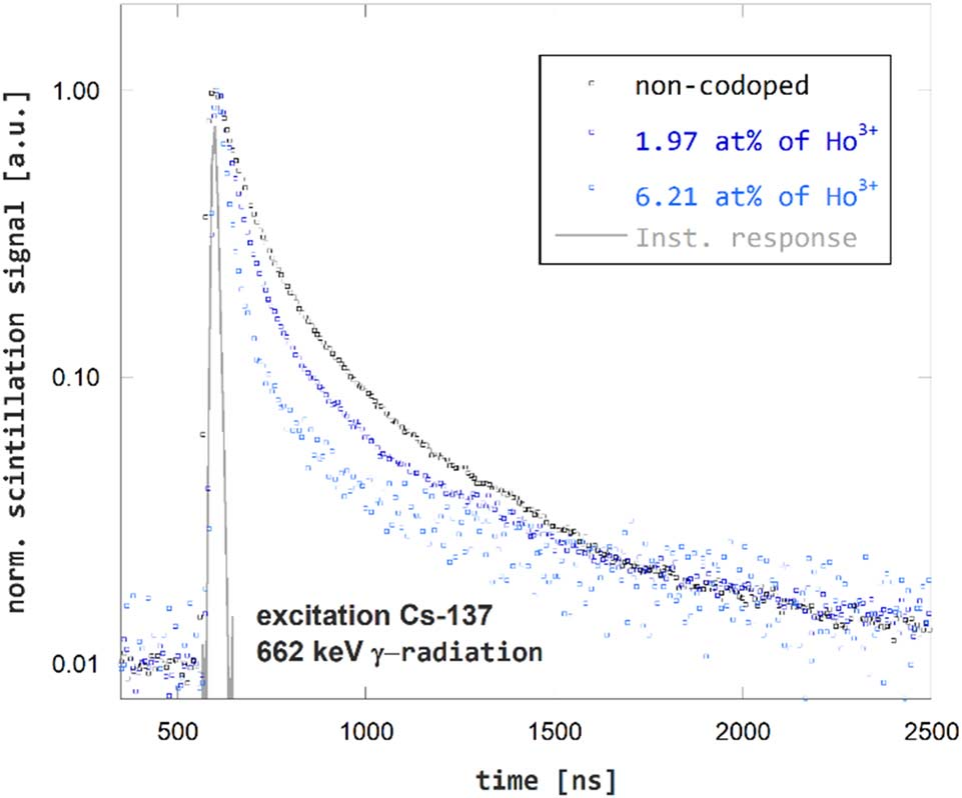
Scintillation decay kinetics of non-codoped GGAG:Ce and Ho^3+^ codoped GGAG:Ce excited by ^137^Cs γ-radiation.

The effect of Ho^3+^-codoping of GGAG:Ce on its scintillation kinetics was studied using time-resolved spectroscopy of scintillation pulses. The scintillation decay curves for non-codoped GGAG:Ce and Ho^3+^ codoped GGAG:Ce crystals are presented in Fig. 5. The effect of shortening Ce^3+^ decay time due to Ho^3+^ codoping is evident in the scintillation response as well. The 1/*e* decay time dropped from 90 ns for non-codoped GGAG:Ce to 25 ns for the GGAG:Ce with 6.2 at% of Ho^3+^ codopant. Refer to Table 1 for 1/*e* scintillation decay times of all examined crystals.

**Fig. 6:**
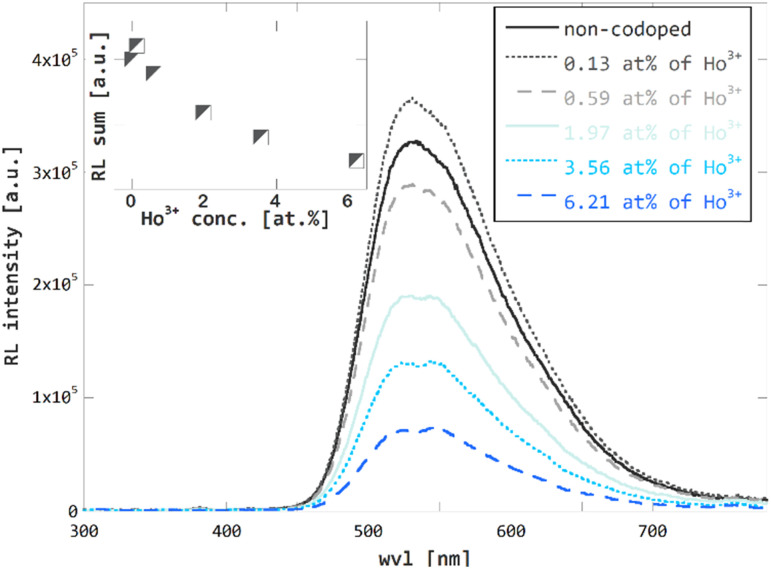
Radioluminescence (40 kV, 15 mA X-rays) spectra of non-codoped GGAG:Ce and Ho^3+^ codoped GGAG:Ce. Radioluminescence spectra integrals plotted against Ho^3+^ codopant concentration in the inset.

RL spectra confirm the expected trend of overall efficiency decrease in Ho^3+^ codoped GGAG:Ce crystals, see the Fig. 6, the concentration dependence of RL spectra integrals in the inset of this figure and Table 1 for listed values of relative overall efficiency (compared to that non-codoped GGAG:Ce). In general, the overall efficiency decreases with Ho^3+^ concentration. For the GGAG:Ce with the highest content of Ho^3+^ codopant the RL spectrum integral drops to 22% of the non-codoped GGAG:Ce. The only deviation from the decreasing trend can be seen for the crystal codoped with 0.13 at% of Ho^3+^ which shows a bit superior RL intensity than the non-codoped GGAG:Ce.

The same crystal deviates from the decreasing trend of scintillation 1/*e* scintillation decay time with value greater by 17% than that of the non-codoped crystal. No such pattern is observed in the trend of 1/*e* photoluminescence decay time or LY measurement, *i.e.* measurements that are mostly determined by the fast components of the scintillation pulses. Due to these facts, the deviation of RL and 1/*e* scintillation decay time is attributed to higher contribution of the slow components, probably due to increased content of traps and defects in the crystal.

In line with our observations from previous studies on Ho^3+^ codoped YAG:Ce^1^ and LuAG:Pr,^3^ no or just negligible Ho^3+^-related emission in the UV-VIS region is observed in RL spectra of Ho^3+^ codoped GGAG:Ce. According to ref. 47, the emission of Ho^3+^ centers in YAG host is located in IR region. The same behavior is anticipated for Ho^3+^ in GGAG. As described above, this makes Ho^3+^ the ideal codopant as it does not introduce any slow components into the detectable light when usual photomultipliers are used as photodetectors. Another advantage of using Ho^3+^ codoping, or RE^3+^ codoping in general, for acceleration of scintillation response using RET in garnet hosts is the expected homogeneous distribution of codopant along the crystal due to very favorable segregation coefficient close to 1.^49^ This ensures rather homogeneous scintillation characteristics in all the volume of the crystal grown. Another, recently published^50^ acceleration mechanism in heavily doped GAGG:Ce,Mg crystals which is based on luminescence quenching in the Ce–Mg pairs, is much more problematic in this respect.

Analogously to RL spectroscopy, the amplitude spectroscopy of scintillation pulses confirms the expected decreasing trend of LY in the Ho^3+^ codoped GGAG:Ce crystals as well. A decrease of LY is proportional to Ho^3+^ concentration. For the highest content of Ho^3+^ LY drops to 15% when compared to that of the non-codoped GGAG:Ce crystal. The data for LY are summarized in Table 1.

**Fig. 7:**
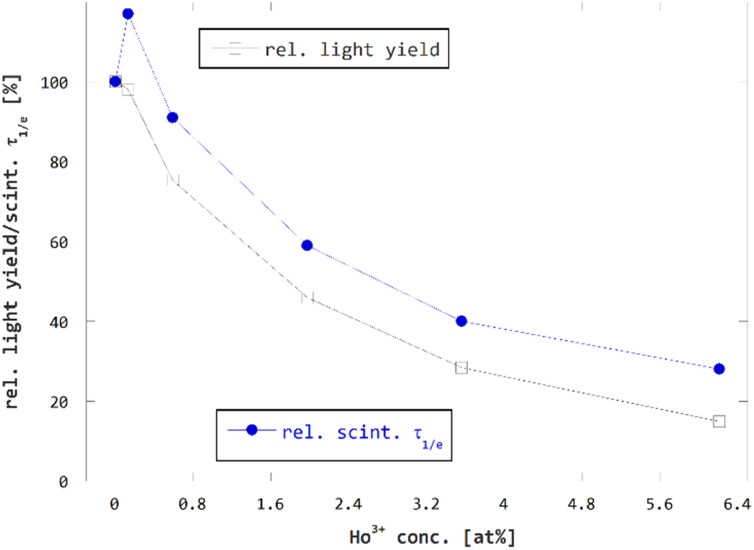
Scintillation 1/*e* decay time *τ*_1/*e*_ and LY of Ho^3+^ codoped GGAG:Ce plotted against Ho^3+^ codopant concentration in relative scale.

The above findings show two effects of Ho^3+^ codoping on scintillation properties of on GGAG:Ce. The first is shortening of the scintillation pulses. In terms of 1/*e* scintillation decay time, the Ho^3+^ codoping can reduce this quantity by tens of percent in GGAG:Ce which improves timing properties of the scintillator, enabling *e.g.* increased detection rate. The second effect of Ho^3+^ codoping is the decrease of scintillator efficiency. In general, decrease of scintillator efficiency is unfavorable as it leads to impaired performance of the material, *e.g.* impaired energy resolution. In terms of LY, the rate of decrease is slightly higher than that of 1/*e* scintillation decay time in GGAG:Ce. One can think of RE^3+^-codoping as a method that enables trading scintillator efficiency for faster scintillation decay. Both measures are put into perspective in Fig. 7 which compares relative LY and scintillation 1/*e* decay time *τ*_1/*e*_. The values are listed in Table 1.

### Energy loss pathways due to Ho^3+^ codoping and their quantification

As shown in the previous section, the Ho^3+^ codoping of GGAG:Ce induces simultaneously an acceleration of scintillation pulses and loss of scintillator efficiency. This is due to interference of Ho^3+^ codopant in multiple stages of the scintillation mechanism. The second part of this study is focused on examination of the origin of losses of scintillator efficiency caused by Ho^3+^ codoping. The main loss pathways are identified and their contribution in overall loss of scintillator efficiency is estimated. The estimations are further compared to the experimental LY data.

We identify following energy loss pathways within the scintillation mechanism of GGAG:Ce caused by Ho^3+^ codoping interferes:

(a) Degradation of the crystal quality due to high concentration of Ho^3+^ codoping. Introduction of new element, especially if introduced in high concentrations can make the crystal growth unstable, introduce new type of defects, and cause overall impairment of the crystal quality.

(b) Charge carriers capture on Ho^3+^ centers during the transport stage of scintillation process. Ho^3+^, same as Ce^3+^ and other RE^3+^ ions, creates recombination centers that capture the electrons and holes during the transport stage of the scintillation conversion mechanism. Once electrons and holes are trapped on Ho^3+^ center, they will slowly deexcite through the dense structure of Ho^3+^ excited states producing photons in IR region, outside detection range of used photosensitive elements of scintillation detectors. As a result, the amount of energy delivered to Ce^3+^ centers and used for generation of detectable scintillation photons is reduced by the part captured on Ho^3+^ codopant and the scintillator efficiency is impaired.

(c) Resonant energy transfer from Ce^3+^ to Ho^3+^. This effect enables shortening of the Ce^3+^ decay time. At the same time, it consumes part of the energy which would be emitted by Ce^3+^ in form of scintillation photons in absence of Ho^3+^ codopant but is resonantly transferred to the Ho^3+^ and consequently emitted in the IR region, *i.e.* technically lost, as described above.

(d) Reabsorption of Ce^3+^ emitted light by Ho^3+^. RET is enabled *via* overlap of Ce^3+^ emission and Ho^3+^ absorption peaks which inevitably enables not only non-radiative (resonant), but also radiative transfer of energy, *i.e.*, part of the Ce^3+^ emitted photons is reabsorbed by Ho^3+^ codopant as shown in Fig. 3.

Assume *l*_*x*_, such as 0 ≤ *l*_*x*_ ≤ 1, is an estimated loss of scintillator efficiency induced due to one of the described energy loss pathways due to Ho^3+^ codoping, and *f*_*x*_ = 1 − *l*_*X*_ is multiplication factor representing scintillator efficiency after accounting the effect of the specific energy loss pathway. Then, if *η*_0_ is the efficiency of the non-codoped crystal, the efficiency of the Ho^3+^ codoped crystal can be estimated as

5 *η* = *f*_deg_*f*_CC_*f*_RET_*f*_reabs_*η*_0_

where deg refers to overall degradation of crystal quality due to codoping, CC refers to charge capture by Ho^3+^, RET to resonant energy transfer from Ce^3+^ to Ho^3+^ and reabs to reabsorption of Ce^3+^ emitted light by Ho^3+^. In the next paragraphs loss of efficiency multiplication factors *f*_*X*_ will be estimated based on experimental data obtained on the non-codoped and Ho^3+^ codoped GGAG:Ce crystals. Finally, an estimate of relative scintillator efficiency can be calculated as6
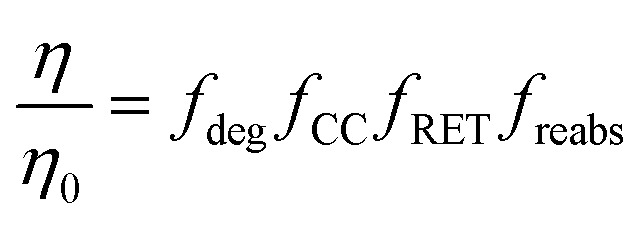


Experimentally obtained values of LY of the non-codoped and Ho^3+^ codoped GGAG:Ce crystals can be used as independent reference to validate estimates of the scintillation efficiency *η*/*η*_0_.

**Fig. 8:**
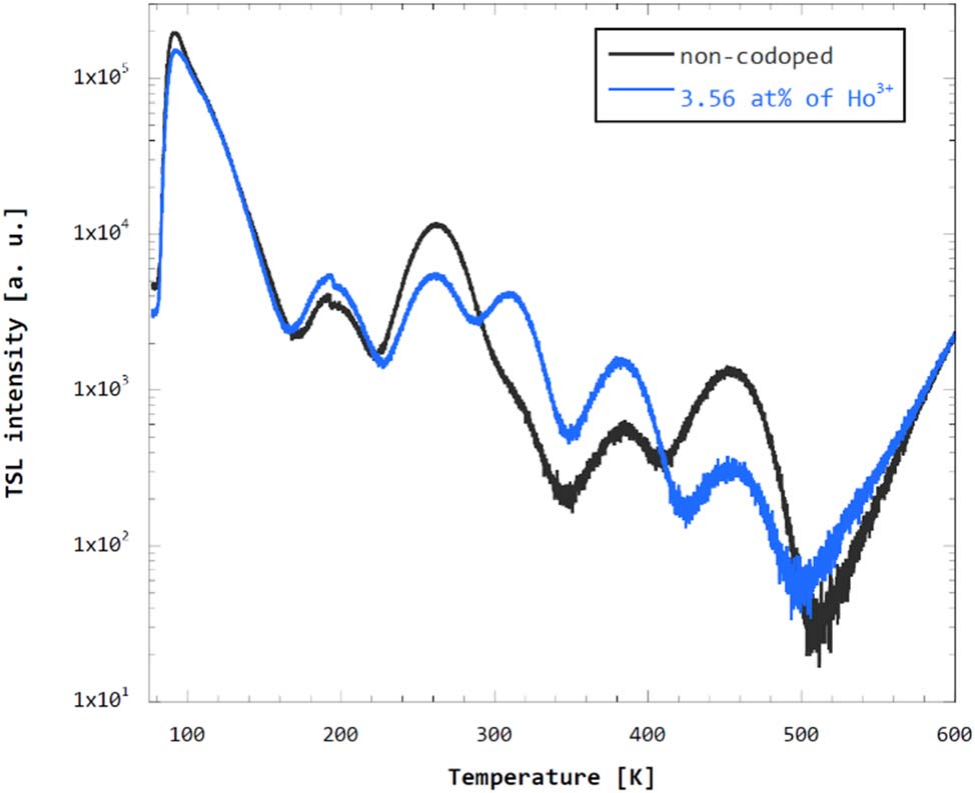
Spectrally unresolved TSL glow curves of non-codoped and Ho^3+^ codoped GGAG:Ce measured after 10 minutes of irradiation with 40 kV/15 mA X-rays at 77 K and 0.1 K s^−1^ heating rate.

To assess the effect of Ho^3+^ codoping of GGAG:Ce on overall crystal quality, mainly impairment due to introduction of new defects due to Ho^3+^ codoping, spectrally unresolved TSL measurement was performed. See Fig. 8 for the glow curve of the non-codoped GGAG:Ce and GGAG:Ce codoped with 3.6 at% of Ho^3+^. Both the glow curves are composed of TSL peaks with the maxima at the same temperatures, although their contribution differs in the non-codoped and Ho^3+^ codoped crystal. No additional TSL peaks referring to a new type of defects due to Ho^3+^ codoping are observed in Ho^3+^ codoped crystal. Therefore, loss of efficiency due to degradation of crystal quality and additional traps is considered negligible and the related multiplication factor *f*_deg_ is set 1 for all Ho^3+^ codoped crystals.

The amount of energy resonantly transferred from Ce^3+^ donor to Ho^3+^ acceptor, *i.e.* the loss of efficiency due to RET, is proportional to a difference of integrals of the Ce^3+^ decay curves in the non-codoped and Ho^3+^ codoped crystal. The multiplication factor *f*_RET_ is then estimated as7
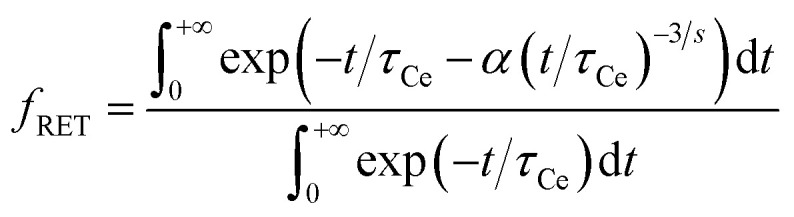
using the results of the curve fitting to IH model. The resulting multiplication factors are listed in Table 2. The estimation shows, the crystal with the highest content of Ho^3+^ loses more than 80% of the efficiency when compared to the non-codoped one due to RET. Based on this result, it is clear, that RET is the main energy loss pathway caused by Ho^3+^ codoping.

**Table 2:** Summary table of factors of efficiency loss for non-codoped GGAG:Ce and Ho^3+^ codoped GGAG:Ce

Ho^3+^ conc. [at%]	*f*_deg_	*f*_CC_	*f*_RET_	*f*_reabs_	*η*/*η*_0_	Rel. LY
0.00	1.00	1.00	1.00	1.00	1.00	1.00
0.13	1.00	1.00	0.94	0.99	0.93	0.98
0.59	1.00	1.00	0.77	0.98	0.76	0.75
1.97	1.00	1.00	0.51	0.97	0.50	0.46
3.56	1.00	1.00	0.32	0.97	0.31	0.28
6.21	1.00	1.00	0.19	0.79	0.15	0.15

To estimate the loss of efficiency due to reabsorption of Ce^3+^ emitted light on Ho^3+^, the obtained photoluminescence spectra, shown in Fig. 3, were used. The loss is proportional to size of the reabsorption dips observed in the photoluminescence spectra of the Ho^3+^ codoped GGAG:Ce. The multiplication factor *f*_reabs_ is estimated as8
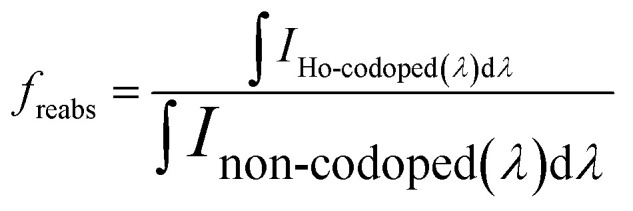
where *I*_Ho-codoped_(*λ*) and *I*_non-codoped_(*λ*) are normalized photoluminescence spectra of the Ho^3+^ codoped and the non-codoped crystal. The spectra were normalized to the values at 580 nm, not to the maxima of the peak, as the maxima is disrupted by reabsorption dips, see Fig. 3. Resulting multiplication factors can be found in Table 2. As reabsorption is given also by length of optical path within the crystal, this estimate is restricted to crystals of the same dimensions as of those used in this study. The losses due to reabsorption of the Ce^3+^ light on Ho^3+^ reach 11% for the crystal with the highest content of Ho^3+^ when compared to the non-codoped crystal.

Summarizing the estimates of efficiency loss due to impaired crystal quality, RET, reabsorption on Ho^3+^ and the relative values of measured LY, we assume the loss of efficiency due to charge carrier capture by Ho^3+^ are very low or negligible.

**Fig. 9:**
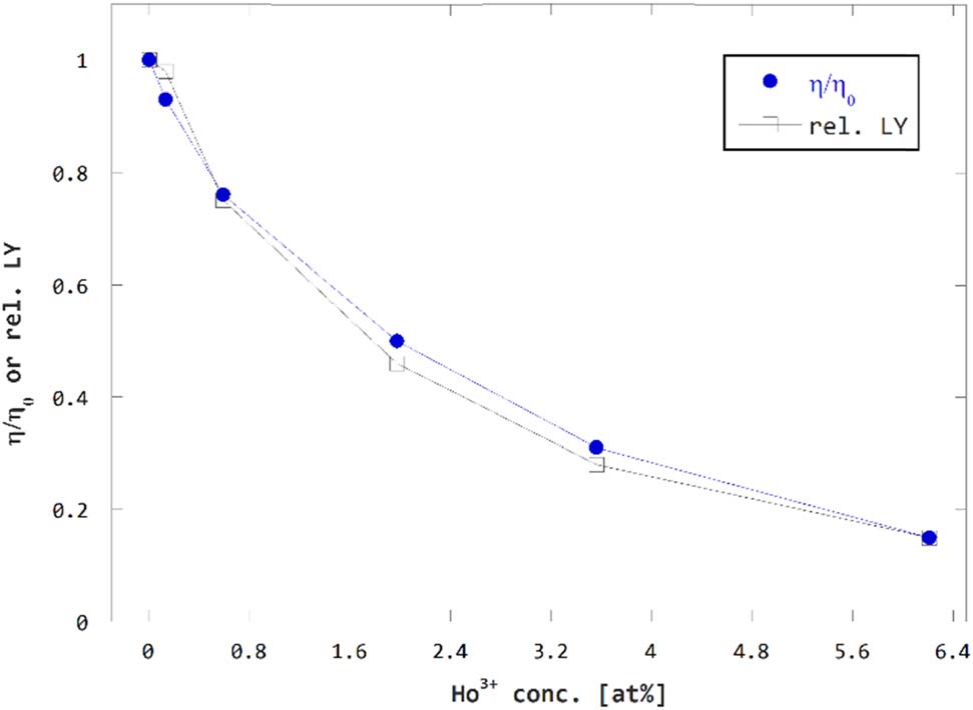
Comparison of concentration dependence of estimated relative efficiency *η*/*η*_0_ and measured relative LY.

In fact, if the multiplicative factor for charge losses due to charge carrier capture *f*_CC_ is assumed to be 1, we obtain a solid match between the resulting estimate of relative efficiency *η*/*η*_0_ and independently measured relative LY values, see Fig. 9. In case, we assumed the loss of efficiency due to charge carrier capture non-zero, *i.e. f*_CC_ < 1, the resulting estimate of relative efficiency *η*/*η*_0_ would only deviate from the experimental LY data. Hence the efficiency losses due to charge carrier capture are considered negligible and related multiplicative factor *f*_CC_ is estimated to 1 for all concentrations of Ho^3+^.

The estimations of losses of scintillator efficiency performed in the previous section helped to reveal more on the impact by Ho^3+^ codoping of GGAG:Ce on specific stages of scintillation mechanism.

The investigation shows by far the greatest part of the losses of scintillator efficiency are due to the RET from Ce^3+^ to Ho^3+^, the same process that is causing the acceleration of Ce^3+^ decay time. A smaller part of losses of scintillation efficiency is due to other means. In other words, most of the lost scintillator efficiency was used for the purpose of the method, *i.e.* acceleration of the activator decay time. This makes Ho^3+^ codoping of GGAG:Ce very effective method for modification of timing properties of scintillation response.

Minor losses of efficiency are caused by reabsorption of Ce^3+^ emitted light by Ho^3+^ centers. The losses might change for a different geometry of a crystal, however, even for large crystals the losses due to reabsorption should saturate at certain level as Ho^3+^ absorption lines are overlapping just a part of the broad emission band of Ce^3+^ and part of the Ce^3+^ emitted light would remain not absorbed. The losses due to reabsorption of Ce^3+^ emitted light on Ho^3+^ do not contribute to shortening of the Ce^3+^ decay time.

Interestingly, the results also suggest that Ho^3+^ does not interfere with charge carriers during the transport stage of scintillation conversion in GGAG:Ce, or, in other words, the Ho^3+^ ability to capture charge carriers is very low when compared to Ce^3+^, even if the concentration of Ho^3+^ is an order of magnitude higher. This could be attributed to Mg^2+^ codoping, that induces formation of Ce^4+^ centers that are more effective in capturing electrons when compared to Ce^3+^ capturing holes.

## Conclusions

In this study the effect of Ho^3+^ codoping on GGAG:Ce luminescence and scintillation properties were investigated with the focus on timing properties and scintillator efficiency and the impact of the Ho^3+^ codoping on different stages of scintillation mechanism of GGAG:Ce. Our results show the Ho^3+^ codoping and the related resonant energy transfer from Ce^3+^ to Ho^3+^ can lead to significant reduction of the Ce^3+^ 5d_1_ excited state decay time and shortening scintillation pulses of GGAG:Ce. At the same time scintillator efficiency is reduced as well. We found the 1/*e* scintillation decay time can be reduced by tens of percent, while light yield decreases by an equivalent amount when using Ho^3+^ codoping in GAGG:Ce.

Moreover, we showed that the Ho^3+^, unlike other RE^3+^as Dy^3+^, Er^3+^ or Nd^3+^, is favorable choice of codopant for the examined method due to absence of slow 4f → 4f emission in the UV/VIS region and thus absence of slow components in the detectable light when using usual photomultipliers.

We showed, the emission kinetics of the Ce^3+^ donor in Ho^3+^ codoped GGAG:Ce can be consistently described with the Inokuti–Hirayama model. Consistency with this model was observed also in our previous studies on various donor–acceptor pairs and matrices. Furthermore, we showed the method provides consistent results for both GGAG and YAG matrix and crystal growth method when doped by Ce^3+^–Ho^3+^ donor–acceptor pairs. Both the consistency with this model and consistency of the results for similar matrices show the effect of RE^3+^ codoping on scintillator properties is reliably predictable.

Further, losses of the scintillator efficiency due to Ho^3+^ codoping were analyzed in the detail. The most significant loss-of-efficiency pathways were identified, and their share on total loss of scintillator efficiency was estimated based on the experimental results. The major losses are attributed to the resonant energy transfer from Ce^3+^ donor to Ho^3+^ acceptor, *i.e.* the same mechanism that shortens Ce^3+^ decay time. Small part of the losses is due to Ho^3+^ reabsorption of Ce^3+^ emission and the losses due other pathways are negligible. Thus, we showed the Ho^3+^ codoping of GAGG:Ce is an effective method for acceleration of its scintillation response, as the largest part of scintillation efficiency losses are due to acceleration itself, not due to effects associated with Ho^3+^ codoping that do not accelerate the scintillation response.

The unprecedented advantage of the examined method is that it can be applied right away on many existing materials. Its use is not limited to garnet matrices or selection of the Ce^3+^–Ho^3+^ donor–acceptor pair, but can be applied to any family of matrices and combination of donor–acceptor pairs that meet the resonance criteria.

## Data availability

Data are available upon request from the corresponding authors.

## Author contributions

Juraj Páterek: conceptualization, formal analysis, investigation, methodology, visualization, writing – original draft; Pavel Boháček: crystal growth, resources, writing – review & editing; Bohumil Trunda: crystal growth, resources; Vladimír Babin, Richard Švejkar, Karel Jurek, Jan Rohlíček: investigation; Martin Nikl: funding acquisition, supervision, writing – review & editing.

## Conflicts of interest

There are no conflicts to declare.

## Supplementary Material

RA-014-D4RA02866J-s001
