# Supplementary material for: Ho3+ codoping of GGAG:Ce: a detailed analysis of acceleration of scintillation response and scintillation efficiency loss
Source: RSC Adv. 2024 Jul 22;14(32):23129–38. doi: 10.1039/d4ra02866j (PMC11262007; doi:10.1039/d4ra02866j)
Supplement: RA-014-D4RA02866J-s001 [file RA-014-D4RA02866J-s001.pdf]

Electronic Supplementary Material (ESI) for RSC Advances.
This journal is © The Royal Society of Chemistry 2024

Ho^{3+} Codoping of GGAG:Ce: A Detailed Analysis of Acceleration of Scintillation Response and Scintillation Efficiency Loss

Juraj Páterek[*a, b], Pavel Boháček[a], Bohumil Trunda[a], Vladimír Babin[a], Richard Švejkar[b], Karel Jurek[a], Jan Rohlíček[a] and Martin Nikl[*a]

[a] Institute of Physics, Czech Academy of Sciences, Cukrovarnická 10, Prague, Czech Republic

[b] Faculty of Nuclear Sciences and Physical Engineering, Czech Technical University in Prague, Břehová 7, Prague, Czech Republic

[*] **Corresponding authors:** paterek@fzu.cz (Juraj Páterek), nikl@fzu.cz (Martin Nikl)

SUPPLEMENTARY INFORMATION

XRD analysis revealed that GGAG:Ce codoped with 6.21 at.% of Ho^{3+} contains two garnet phases, see Figure S1. The secondary phase copies the XRD pattern of the primary one, however, the peaks are shifted towards lower angles. These results suggest the that both phases are structurally similar, but the secondary phase has larger unit cells. The amount of the secondary phase is estimated up to 5 wt%. Any additional phase was not found in other samples, see Figure S2 with XRD spectrum of the non-codoped GGAG:Ce.

The secondary phase island in GGAG:Ce codoped with 6.21 at.% of Ho^{3+} was localized using back-scattered electrons (BSE) imaging, see Figure S3, and further analyzed with EPMA. The results showed the content of Ho^{3+} in secondary phase was reduced to one third when compared to the primary phase in favor of Gd^{3+}. This is in line with the observations from XRD, as garnet structure with higher amount of Ho^{3+} are expected to have smaller unit cell as the lattice parameter for $Gd_3Al_5O_{12}$ is 12.21 Å while that of $Ho_3Al_5O_{12}$ is 12.08 Å (1).

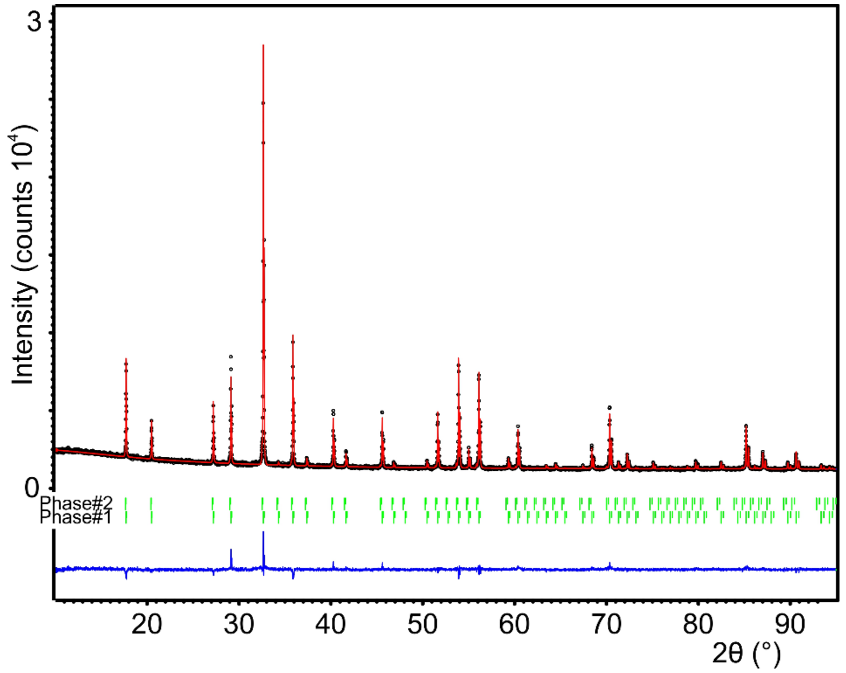

Figure S1: Result of Rietveld refinement of GGAG:Ce codoped with 6.21 at.% of Ho^{3+}. The black points represent the measured pattern, the red line corresponds to the calculated pattern, the blue curve is the difference between the measured and calculated data, and the green bars show the Bragg positions of both phases.

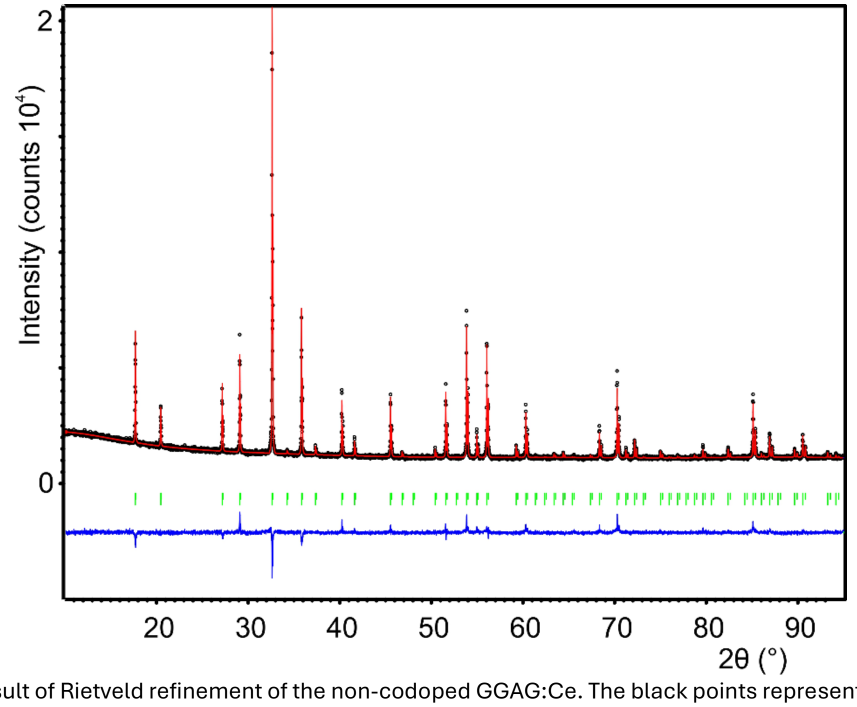

Figure S2: Result of Rietveld refinement of the non-codoped GGAG:Ce. The black points represent the measured pattern, the red line corresponds to the calculated pattern, the blue curve is the difference between the measured and calculated data, and the green bars show Bragg positions.

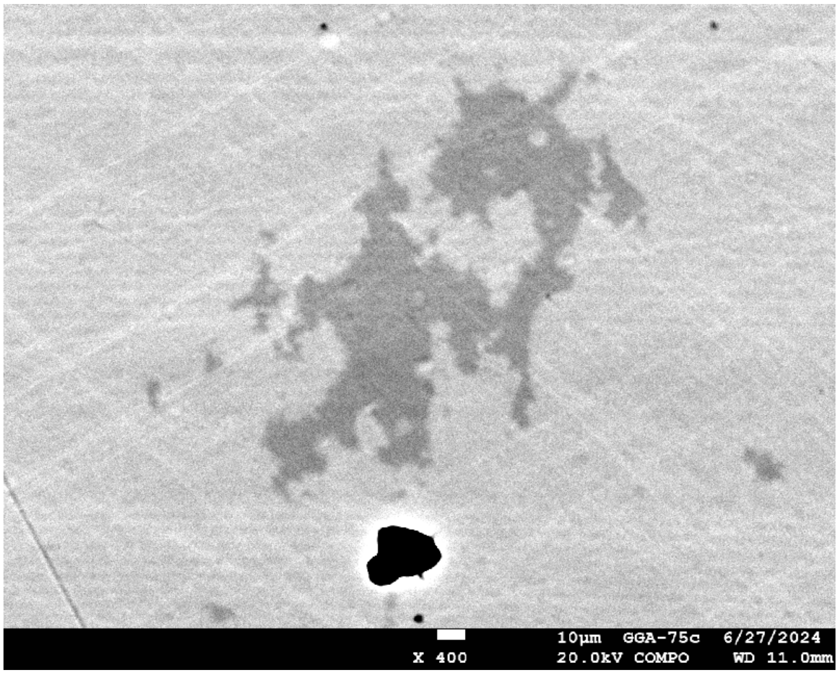

Figure S3: BSE image of secondary phase (light grey islands) within GGAG:Ce codoped with 6.21 at.% of Ho^{3+}. Black spot in the central bottom is an instrumental effect.

REFERENCES

1. Jain A, Ong SP, Hautier G, Chen W, Richards WD, Dacek S, et al. Commentary: The Materials Project: A materials genome approach to accelerating materials innovation. APL Materials. 2013 Jul;1(1):011002.
